# Organ‐specific endothelial heterogeneity: From developmental blueprints to precision vascular medicine

**DOI:** 10.1002/ctm2.70820

**Published:** 2026-09-11

**Authors:** Yu‐xiang Wang, Qi Chen, Yang Liu

**Affiliations:** ^1^ Center for Cell Lineage Atlas Guangzhou Institutes of Biomedicine and Health Chinese Academy of Sciences Guangzhou China; ^2^ Key Laboratory of Digital Life and Medicine Guangdong Provincial Key Laboratory of Stem Cell and Regenerative Medicine Guangdong‐Hong Kong Joint Laboratory for Stem Cell and Regenerative Medicine Guangzhou Institutes of Biomedicine and Health Chinese Academy of Sciences Guangzhou China; ^3^ University of Chinese Academy of Sciences Beijing China; ^4^ The Innovation Centre of Ministry of Education for Development and Diseases School of Medicine South China University of Technology Guangzhou China

1

For decades, endothelial cells (ECs) have been widely believed as a resting, uniform cell layer covering the entire circulatory system. However, this traditional view has been largely challenged over the last decade. Early microarray studies showed that microvascular ECs isolated from different organs display distinct molecular profiles. These ECs express unique combinations of transcription factors, adhesion molecules and chemokines exhibiting variable regenerative capacity after tissue injury.^1^

Based on this important finding together with other implications, Augustin and Koh proposed the key concept of ‘organotypic vasculature’ in 2017, representing a major theoretical advance in vascular biology.^2^ This review re‐defined the understanding of endothelial heterogeneity. Instead of reflecting simple structural differences among blood vessels, EC heterogeneity is now recognised as a core functional mechanism controlling tissue homeostasis, metabolic balance, inflammation and tumour progression.^2^ The conceptual progress promotes potential translational research in this field, encouraging researchers to move beyond broad vascular protective strategies and focus on the rational design to precisely target ECs in each organ.

The rapid development of single‐cell RNA sequencing technology (scRNA‐seq) enabled accurate molecular analysis of EC heterogeneity across different organs. In 2020, Kalucka and colleagues analysed over 32 000 ECs from 11 mouse tissues and identified a large number of distinct EC subsets. Their work clearly confirmed that the residing tissue is one of the primary factors distinguishing endothelial heterogeneity.^3^ In parallel, Paik and colleagues used scRNA‐seq to define core transcriptional signatures that distinguish ECs from different organs.^4^ While these mouse atlases provide essential reference data for endothelial research, their clinical relevance requires further validation in human tissues. A study in 2024 filled this critical research gap by generating a comprehensive human vascular cell atlas. This atlas includes approximately 67 000 vascular cells collected from 19 organs across 62 adult donors and identifies 42 distinct vascular cell states.^5^ The work confirms that organ‐specific endothelial heterogeneity also exists among different organs of adult humans. However, it also reveals human‐specific inflammatory and regulatory features that cannot be detected in rodent models. For example, the key pulmonary transcription factor *Foxf1* shows distinct expression patterns in human and mouse lung vascular subpopulations, highlighting the importance of human tissue research for clinical translation.^5^

These early endothelial atlases mainly describe the static molecular characteristics of adult ECs. Our recent study clarifies the emergence timing of organ‐specific endothelial features.^6^ The organ‐specific EC identities are not acquired after birth; instead, they are gradually established through strict genetic regulation during embryonic development. Our high‐resolution time‐series transcriptomic dataset improves this understanding (Figure 1). Systematic analysis of 26 continuous embryonic time points across eight organs shows that ECs initiate organ‐specific differentiation as early as embryonic day 8, with continuous molecular and functional maturation throughout mid‐gestation of mouse.^6^ Importantly, functional studies of *Casz1*, a transcription factor highly enriched in lung ECs, move beyond descriptive transcriptomic analysis to reveal causal regulatory roles. The results based on endothelial‐specific knockout of *Casz1* support that removal of *Casz1* blocks establishment of lung ECs organ specificity. Therefore, *Casz1* is essential for pulmonary vascular development, organ‐specific EC differentiation, and signal communication between endothelial and epithelial cells.^6^ Thus, the organ‐specific endothelial heterogeneity establishes in embryonic stage and persists into adulthood in homeostasis. However, the organotypic vascular zonation can be damaged under pathological condition. For example, the liver sinusoidal endothelial cells (LSEC) eliminating the unique molecular signature with decreased expression of LSEC‐specific transcription factors *c‐* *Maf* and *Gata4* in liver cirrhosis, are substituted by pan‐endothelial cells, accompanied by impaired hepatic material transport and metabolic regulation.^7^

**FIGURE 1:**
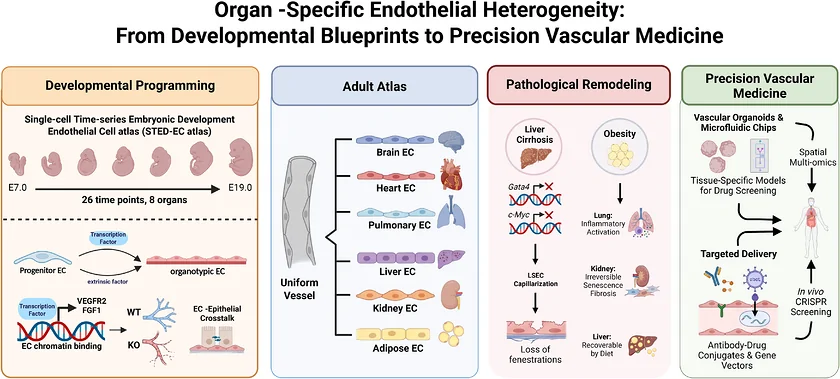
Schematic representation of organ‐specific endothelial heterogeneity: developmental programming, pathological divergence and targeted translational vascular therapeutics.

Furthermore, endothelial heterogeneity strongly influences vascular responses under pathological conditions, especially in systemic metabolic diseases. Large‐scale single‐cell analysis of over 375 000 ECs from seven organs shows that obesity causes progressive, organ‐ and subtype‐specific changes in endothelial lipid metabolism, energy balance and AP1‐dependent inflammatory signalling.^8^ Specifically, pulmonary ECs tend to undergo strong inflammatory activation under metabolic stress, while renal ECs are prone to irreversible cellular senescence. Notably, dietary intervention effectively rescues transcriptional and functional defects in hepatic ECs but has minimal protective effects on senescent renal ECs.^8^ This obvious difference suggests that the divergent endothelial responses to systemic pathological stress are intrinsically determined by developmental programming and organ‐specific physiological properties. These developmental and functional differences explain the inconsistent and limited effects of conventional generalised vascular treatments across different organs, strongly supporting the development of tailored organ‐specific therapeutic strategies.

Two research directions may promote translational progress in this field. Firstly, developmental endothelial atlases support the in vitro generation of tissue‐matched vascular structures. During embryonic development, ECs undergo functional diversification of angiocrine signalling, which endows organ‐specific ECs with unique regenerative ability and microenvironmental regulatory functions.^9^ By reconstructing core developmental transcription factor networks using vascular organoid models and microfluidic platforms, researchers may generate stable, tissue‐specific vascular units. These engineered vascular systems overcome the limitations of traditional uniform EC culture models and provide reliable tools for high‐throughput drug screening and disease mechanism research.^9^

Secondly, organ‐specific molecular markers and core regulatory pathways identified from human vascular atlases facilitate the development of precise vascular therapies. Targeted delivery systems, including antibody‐drug conjugates and endothelial‐specific gene vectors, can selectively target damaged vascular tissues, preserve normal systemic vascular function, and reduce non‐specific treatment side effects. Advanced technologies, such as spatial multi‐omics and in vivo CRISPR functional screening, drive the field from descriptive cell profiling to causal mechanism research, enabling the identification of clinically useful therapeutic targets.^10^

In summary, research on endothelial organ‐specific heterogeneity has progressed from basic morphological observation and molecular profiling to in‐depth analysis of developmental and pathological mechanisms, and is now advancing toward precise clinical application. Integrating developmental regulatory patterns and disease susceptibility provides a new framework for understanding the diverse pathological features of vascular diseases. Future studies focusing on core mechanisms will support the development of accurate organ‐targeted vascular therapies, improving the treatment of refractory vascular diseases with prominent organ‐specific pathological characteristics.

## CONFLICT OF INTEREST STATEMENT

The authors have declared that no conflict of interest exists.

## FUNDING INFORMATION

This work is supported by the National Key R&D Program of China (2024YFA1802200), National Natural Science Foundation of China (32270866, 32300693) and China Postdoctoral Science Foundation (GZC20261503).
